# Should we prescribe anticonvulsants for acute herpes zoster neuralgia and to prevent postherpetic neuralgia? A protocol for meta-analysis and benefit-risk assessment

**DOI:** 10.1097/MD.0000000000024343

**Published:** 2021-02-19

**Authors:** Yanqing Lu, Kun Liu, Yanchang Liang, Xi Zhang, Yue Liu, Fan Huang, Haili Gao, Lixing Zhuang

**Affiliations:** a Guangzhou University of Traditional Chinese Medicine; b Guangzhou University of Traditional Chinese Medicine, Fifth Clinical Medical College; c Guangzhou University of Traditional Chinese Medicine, Sports and Health Institute; d Guangzhou Hospital of Traditional Chinese Medicine, Neurosurgery Department; e Guangzhou University of Traditional Chinese Medicine First Affiliated Hospital, Acupuncture Department, Guangzhou, Guangdong, China.

**Keywords:** acute herpes zoster neuralgia, anticonvulsants, postherpetic neuralgia

## Abstract

**Background:**

Herpes zoster-associated pain [i.e., acute herpes zoster neuralgia (AHN) and postherpetic neuralgia (PHN)] has the potential to cause significant patients’ burden and heath resource expenditure. PHN is refractory to the existing treatments, and the consensus is preventing the transition of AHN to PHN is better than treating PHN. Anticonvulsants (e.g., gabapentin, pregabalin) have been recommended as one of the first-line therapies for PHN. In practice, anticonvulsants have also decreased the severity and duration of AHN and reduced the incidence of PHN. Nevertheless, its clinical application to AHN is hampered by inadequate evidence for its efficacy and safety. We performed this protocol for a systematic review to explore the efficacy and safety of anticonvulsants for AHN. Besides, a benefit-risk assessment of anticonvulsants for AHN would be performed to estimate the extent to which these drugs could relieve symptoms and whether the benefits outweigh harms.

**Methods:**

The Preferred Reporting Items for Systematic Review and Meta-Analysis Protocols (PRISMA-P) was used to prepare our protocol and the results will be reported according to the PRISMA. We will search the China National Knowledge Infrastructure (CNKI), Chinese VIP Information (VIP), Cochrane Library, Embase, and PubMed databases, from inception to August 2019. Furthermore, Clinicaltrials (http://www.clinicaltrials.com) and Chinese Clinical Trial Registry (http://www.chictr.org.cn/abouten.aspx) will also be searched for relevant studies. Selection of eligible articles and data extraction will be independently performed by reviewers. We will record the characteristic information, pain outcomes, incidence of PHN and adverse effects. Data synthesis and other statistical analyses will be conducted using Review Manager Software 5.3 and STATA13.0. Furthermore, risk of bias assessment, meta-regression and subgroup analyses, publication bias assessment, grading of evidence will be performed for included studies.

**Ethics and dissemination:**

As this systematic review will be performed based on published data, no ethical approval is needed. The findings will be submitted in peer-reviewed journals for publication.

**Systematic review registration:**

PROSPERO CRD42019133449.

## 1 Introduction

Herpes zoster, caused by reactivation of the dormant varicella zoster virus in a sensory ganglia, is featured by unilateral distribution of rash and pain.^[1]^ Severe zoster-associated pain, including both acute herpes zoster neuralgia and postherpetic neuralgia,^[2]^ has the potential to significantly lower patient's quality of life and even limit daily activities.

Zoster-associated pain results from viral damage and increased sensitization of affected segmental sensory neuron.^[3]^ Pain during the first 30 days of onset is known as acute herpetic neuralgia (AHN).^[4]^ Postherpetic neuralgia (PHN), defined as pain persisting for over 1 month after the healing of rash,^[5,6]^ is one of the commonest neurologic complications of herpes zoster. In recent guidelines, Neuropathic Pain Special Interest Group have recommended gabapentin, pregabalin, duloxetine, venlafaxine, and tricyclic antidepressants as first-line therapy^[7,8]^ for PHN, of which gabapentin and pregabalin have been widely used. Besides, topical therapy and various other attempts such as epidural blocks, sympathetic nerve blocks, and stellate ganglion pulsed radiofrequency stimulation and these have proved to reduce PHN. Despite combined therapies, only less than 50% of patients have achieved a significant reduction (>50%) in pain.^[8,9]^

The consensus is that prevention is better than treatment. Recent years have seen substantial efforts to identify preventive measures for PHN. However, effective methods with strong evidence are limitted only to vaccinations, nerve blocks and some other supplemental therapies for now.^[10–12]^ No adequate evidence shows that oral drugs in the acute phase could significantly reduce the incidence of PHN. There is high-quality evidence to show that oral aciclovir fails to significantly reduce the incidence of PHN.^[13,14]^

Recently, more attention has been focused on the pain management in the acute phase of herpes zoster.^[15,16]^ The prevention strategy of avoiding the transition of AHN to PHN is in accordance with the international perception concerning chronic pain management.^[17]^ As for acute AHN, clinical guidelines generally recommend the 3-step WHO pain ladder.^[18,19]^ Combinations of NSAIDs or other nonopioids and, nonopioids plus weak opioid analgetics or strong opioids vary according to the severity of pain. Considering the neuropathic component of the acute pain, anticonvulsants (e.g., gabapentin, pregabalin) may be used as supplements to the basic analgesia treatment.^[17]^ Recently, anticonvulsants alone or combined with other drugs have been found to have significant beneficial effects,^[20–22]^ however, inadequate evidence have been identified. In fact, controversy over the effectiveness and safety of anticonvulsants in preventing PHN remains fierce,^[4,23]^ the same being the case with their effects on acute herpes zoster neuralgia.

## 2 Objectives

This protocol is designed to establish a systematic review and meta-analysis, including all the available studies reported in Chinese and English, to determine the efficacy and safety of anticonvulsants on the analgesia of acute herpes zoster neuralgia and the prevention of PHN.

## 3 Methods and analysis

### 3.1 Registration and review design

This protocol has been developed according to the Cochrane Back Review Group guidelines, the Cochrane Handbook, and the Preferred Reporting Item for Systematic Reviews and Meta-Analyses Protocols (PRISMA-P) (see Additional file 1).^[24–27]^ This protocol has been registered on PROSPERO (CRD42019133449) on the August 14, 2019.

### 3.2 Search strategy

We will perform electronic database searches on China National Knowledge Infrastructure (CNKI), Chinese VIP Information (VIP), Cochrane Library, Embase, and PubMed databases, from inception to August 2019. Furthermore, Clinicaltrials (http://www.clinicaltrials.com) and Chinese Clinical Trial Registry (http://www.chictr.org.cn/abouten.aspx) will be searched for ongoing trials. We developed a search strategy using a combination of medical subject headings terms and text words, including the following terms “anticonvulsants” “antiepileptic” “herpes zoster” “clinical trial ”, etc. Modification for keywords and syntax are required to reflect differences of specific search terms between databases, with the search strategy consistent. Full search terms are shown in Additional file 2. In addition, we will also search reference lists of eligible studies and related systematic reviews to determine potentially relevant studies. Only the most updated and recent reports of studies will be included in the final analysis if duplicate publications exist.

### 3.3 Inclusion and exclusion criteria

The population, intervention, comparison, outcomes strategy has been used to formulate our eligibility criteria.^[25,27,28]^

### 3.4 Population

To be eligible, patients undergoing AHN (≦30 days of onset)^[4]^ will be included, without limitations of ages, races, or VAS baselines, etc.

### 3.5 Intervention

The intervention of interest is the administration of anticonvulsants (as classified by the World Health Organization (WHO) Collaborating Centre for Drug Statistics Methodology, N03A Antiepileptics)^[29]^ for relieving pain in the acute phase, irrespective of route (e.g., oral, topical, or intravenous injection), dose and duration. We will exclude studies investigating anticonvulsants administered in the PHN phase or for unclear neuropathic pain.

### 3.6 Comparison

The control intervention could be a placebo or active comparator (other analgesic medications or antiviral drugs only), and within-groups or between-groups.

### 3.7 Outcomes

As for outcomes, studies should report results of pain intensity in last 24 hours or just at that moment (e.g., visual analogue scale, 11-point Likert scale, numerical rating scale, or other measuring methods) as the primary outcome.

The secondary outcomes will also be included, such as the incidence of PHN, adverse events, etc. We will put studies into subgroups by primary outcomes when performing meta-analyses.

### 3.8 Studies

In accordance with the Cochrane handbook,^[25]^ the highest level of evidence for this systematic review shall comprise of randomized controlled trials (RCTs). We will search for prospective RCTs, excluding crossover designs, N-of-1 trials, cohort studies, quasi-RCTs, etc. There are no restrictions of languages or publications in searching process. However, due to limitations in translation tools and time, studies not in English or Chinese will be excluded, but noted on the PRISMA flow diagram.

### 3.9 Selection of studies

Two reviewers (Yanqing Lu, Kun Liu) will respectively screen titles and abstracts of the search results. Another reviewer (Haili Gao) will be consulted in case of any uncertainty. Screening full-text articles for eligibility will be performed independently by 2 reviewers (Kun Liu, Yanchang Liang). Disagreements will be resolved by discussion first, then by arbitration of a third reviewer (Xi Zhang) if needed.

### 3.10 Data extraction and management

After independently screening and evaluating eligible full-texts, 2 reviewers (Yue Liu, Fan Huang) will extract information as the follows: the first author, publication year, sample size, dose and schedule, duration, outcomes, efficacy assessments, and adverse effects, using the tool of Microsoft Excel 2019. As for pain outcomes, we will extract data at the final assessment reported in the course of treatment, with end points varying from the 2nd week to the 4th week, according to the durations. If there are several follow-ups reported, we will record the number of participants with occurrence of PHN at the first follow-up. Furthermore, the reported incidence of any serious or common adverse events will be recorded by types.

If data is missing from studies or presented in an ambiguous way, we will try to contact the corresponding authors for clarification. Any relevant data obtained by clarification will be included into our extraction. If no further information is supplemented in this manner, the study should be considered ineligible and discarded from our systematic review.

### 3.11 Risk of bias

Two reviewers (Haili Gao, Lixing Zhuang) will independently assess the risk of bias (RoB) of individual studies using the Cochrane risk of bias (RoB 2.0) tool for randomized trials,^[30,31]^ with disagreements resolved by consensus. Bias is assessed in 5 distinct domains: randomization process; deviations from intended interventions; missing outcome data; measurement of the outcome; selection of the reported result. Within each domain, we will answer 1 or more signalling questions to a proposed RoB judgment for each domain as “low risk of bias”, “some concerns”, or “high risk of bias”. These judgments will lead to an overall RoBjudgment for the result.^[31]^

### 3.12 Data synthesis and statistical analysis

We will conduct the statistical analysis using Review Manager Software, Version 5.3 and STATA, Version 13.0. Continuous data will be calculated using mean differences (MDs; if the same scale was applied) with 95% confidence intervals (95% CIs) or standardized mean differences (SMDs; if different scales were used)^[32]^ with 95% CIs. Risk ratios (RRs) will be calculated for dichotomous data during synthesis (incidence of PHN and adverse events). A 2-tailed *P* value <.05, which corresponds to the merger effect values, is predetermined for statistical significance.^[33,34]^

### 3.13 Assessment of heterogeneity

Statistical heterogeneity will be tested by the *I*^2^ statistic and Cochran's Q statistic.^[35]^*I*^2^ < 50% and *P* values >.10 are assumed to be no statistically significant heterogeneity and a fixed-effects model will be used to obtain a pooled RR, MD, or SMD with 95% CI. A random-effects model will be utilized if heterogeneity is acceptable (e.g., 50% ≦ *I*^2^ ≦ 75%). The *I*^2^ > 75% is regarded as statistical high heterogeneity and pooling will not be conducted.^[36]^

### 3.14 Metaregression and subgroup analyses

If a statistical high heterogeneity is present, we will perform a metaregression analysis to help identify the potential sources for heterogeneity. Considering demographic and clinical characteristics, several variables will be predetermined to put into analysis, such as the race and gender of the subjects, oral method (e.g., single dosing; titration up to maximal dose), average age (e.g., ≦65; >65), male ratio (e.g., ≦50%; >50), treatment duration (e.g., 2 weeks, 3 weeks, 4 weeks), etc. Moreover, the category of drugs (e.g., gabapentin, pregabalin, or other anticonvulsants) and the languages in which studies are reported will also be considered into metaregression analysis.

First, univariate metaregression models will be used to explore the moderating effects of predetermined variables on the MDs or SMDs, using Q-statistics.^[37]^ Variables in mean estimates at *P* < .2 in our univariate metaregression models will be considered as indicators of heterogeneity, and will subsequently be put into multivariate metaregression models. After calculations,^[38,39]^ variables with a *P* < .05 will be identified as the most influential moderators contributing to heterogeneity, based on which subgroup analyses will be performed. Otherwise, we will conduct a descriptive analysis if no moderators are found.^[40]^ Two reviewers (Yanqing Lu, Kun Liu) will independently perform the metaregression and subgroup analyses, and all disagreements will be discussed until consensus is reached.

### 3.15 Publication bias

We will assess and quantify publication bias using a funnel plot, Begg rank correlation and Egger regression test.^[41,42]^ Trim and fill method^[42]^ will be used to reappraise the effects of publication bias and adjust the pooled HRs, if a < 0.10 *p* Egger exists.^[43,44]^ Funnel plots will be used to report publication biases if ≧10 studies are included in our meta-analysis.^[45]^

### 3.16 Grading of evidence

We will evaluate the overall quality of evidence using a Grading of Recommendations Assessment, Development and Evaluation (GRADE) approach,^[46–48]^ which offers instructions and guidances to rate the quality of evidence for each outcome in a systematic review and categorize them into high, moderate, low, or very low. Two assessors (Yanchang Liang, Haili Gao) will independently assess each outcome in our review according to the GRADE criteria of RoB, indirectness, imprecision, inconsistency, publication bias. We will use the GRADE’ s official GRADEpro software tool (www.gradepro.org/) to produce GRADE evidence summary tables, in which summary statistical information and assessments of certainty are demonstrated. Discussions will be used to resolve any disagreement and the third assessor (Lixing Zhuang) will make a decision.

### 3.17 Benefit-risk assessment

Metric indices including the “number needed to treat to benefit” (NNTB) or to harm (number needed to treat to harm (NNTH)), and the “likelihood to be helped or harmed” will be used in our benefit-risk assessment. We will use the following formula to calculate NNTB/NNTH values: NNTB/NNTH = 1/RD. The “risk difference” (RD) is a widely used absolute effect measure to show the effect of an intervention or an exposure compared with a control group.

## Acknowledgments

We would like to acknowledge the funders in our work.

## Author contributions

**Conceptualization:** Yanqing Lu, Kun Liu, Yue Liu, Lixing Zhuang.

**Data curation:** Yanqing Lu, Kun Liu, Yanchang Liang.

**Formal analysis:** Yanqing Lu.

**Investigation:** Xi Zhang.

**Methodology:** Xi Zhang.

**Project administration:** Haili Gao.

**Resources:** Yue Liu.

**Software:** Haili Gao.

**Supervision:** Haili Gao.

**Validation:** Fan Huang.

**Visualization:** Fan Huang.

**Writing – original draft:** Yanqing Lu, Kun Liu, Lixing Zhuang.

**Writing – review & editing:** Yanqing Lu, Kun Liu, Lixing Zhuang.
